# Supplementary material for: RFRP-3 Influences Apoptosis and Steroidogenesis of Yak Cumulus Cells and Compromises Oocyte Meiotic Maturation and Subsequent Developmental Competence
Source: Int J Mol Sci. 2023 Apr 10;24(8):7000. doi: 10.3390/ijms24087000 (PMC10138887; doi:10.3390/ijms24087000)
Supplement: Supplementary file 1 [file ijms-24-07000-s001.zip › ijms-2248301-supplementary.docx]

Table S1 The sequences of primers and gene accession number used in RT-qPCR.

| Gene | Primer sequence | Gene accession number |
| --- | --- | --- |
| *GnIH* | Fw: TTGGGAAGTCAGTGCCCATC  Rs: CACGGTGCATCTTTTCTGGG | XM_015853673.2 |
| *GPR147* | Fw: CACGAGATACATGCAGGCGA  Rs: GTTTGTCAAGCCCACCACAC | NM_022146.5 |
| *Cdc42* | Fw: GCAATATTGGCTGCCCTGGA  Rs: CAAAAAGGGCTCTGGAAAGGC | FJ358601.1 |
| *Ccnd1* | Fw: CAGACCTTCGTTGCCCTCTG  Rs: CGGGTCACATCTGATCACCTT | KY420723.1 |
| *Pcna* | Fw: GACATCAGCTCAAGTGGCGT  Rs: GTCTTCATTGCCAGCACATTTT | NM_001034494.1 |
| *Caspase-3* | Fw: AGAAGATACCGGTGGAGGCT  Rs: GTGGAAAGTGGAGTCCAGGG | NM_009810.3 |
| *Bax* | Fw: GACTCTCCCCGAGAGGTCTT  Rs: GTCCAATGTCCAGCCCATGA | XM_005895371.1 |
| *Bcl-2* | Fw: CCTGTGGATGACCGAGTACC  Rs: CAGACTGAGCAGTGCCTTCA | MK050976.1 |
| *LHR* | Fw: CGTTGGAGAAACACAGTGCG  Rs: TCCATGAGCAGCAAGTCGTT | NM_137366.4 |
| *StAR* | Fw:AGCTCATACTAAAGGAGCCGTG  Rs: AAAGATTCCAGTAACATCCTAC | NM_174189.3 |
| *3β-HSD* | Fw: CGGGTGCTAGACAAAGTCTTC  Rs: GTGTGGATGACCACTGAGGTG | XM_027535608.1 |
| *Gapdh* | Fw: GGGTCATCATCTCTGCACCT  Rs: GGTCATAAGTCCCTCCACGA | XM_027541122.1 |

Fw: forward, Rs: reverse.
